# Art and Perception: Using Empirical Aesthetics in Research on Consciousness

**DOI:** 10.3389/fpsyg.2022.895985

**Published:** 2022-06-09

**Authors:** Ulrich Ansorge, Matthew Pelowski, Cliodhna Quigley, Markus F. Peschl, Helmut Leder

**Affiliations:** ^1^Faculty of Psychology, University of Vienna, Vienna , Austria; ^2^Vienna Cognitive Science Hub, University of Vienna, Vienna, Austria; ^3^Research Platform Mediatised Lifeworlds, University of Vienna, Vienna, Austria; ^4^Department of Behavioural and Cognitive Biology, University of Vienna, Vienna, Austria; ^5^Department of Philosophy, University of Vienna, Vienna, Austria

**Keywords:** aesthetics, art, consciousness, perception, vision, empirical aesthetics

## Abstract

Understanding consciousness is a major frontier in the natural sciences. However, given the nuanced and ambiguous sets of conditions regarding how and when consciousness appears to manifest, it is also one of the most elusive topics for investigation. In this context, we argue that research in empirical aesthetics—specifically on the experience of art—holds strong potential for this research area. We suggest that empirical aesthetics of art provides a more exhaustive description of conscious perception than standard laboratory studies or investigations of the less artificial, more ecological perceptual conditions that dominate this research, leading to novel and better suited designs for natural science research on consciousness. Specifically, we discuss whether empirical aesthetics of art could be used for a more adequate picture of an observer’s attributions in the context of conscious perception. We point out that attributions in the course of conscious perception to (distal) objects versus to media (proximal objects) as origins of the contents of consciousness are typically swift and automatic. However, unconventional or novel object-media relations used in art can bring these attributions to the foreground of the observer’s conscious reflection. This is the reason that art may be ideally suited to study human attributions in conscious perception compared to protocols dedicated only to the most common and conventional perceptual abilities observed under standard laboratory or “natural”/ecological conditions alone. We also conclude that art provides an enormous stock of such unconventional and novel object-media relations, allowing more systematic falsification of tentative conclusions about conscious perception versus research protocols covering more conventional (ecological) perception only. We end with an outline of how this research could be carried out in general.

## Introduction

Consciousness is an extremely important research topic and one of the last great frontiers in the natural sciences (e.g., Chalmers, 1996; Melloni et al., 2021). In the current context, when we speak of consciousness we refer to the subjective experience from a first-person perspective that is only directly accessible to us, the experiencing subjects. It is about our feelings of pain or pleasure, about how we experience a color or smoothness, in sum about what has been labeled as *qualia* (Nagel, 1974; Dennett, 1988). Think of a time when you were in pain. Think of how it felt, how you were aware of it, how this awareness might have changed, how it manifested in your perceptions, sensations, your awareness of your body, maybe how you wished to not be conscious or to be sedated. These qualia are the fundaments of what it means to be conscious.

For centuries, scientists continue to reflect upon and to seek to understand consciousness (e.g., Hegel, 1807; Descartes, 2011). Today, natural sciences are increasingly seeking to help us find solutions to this problem (e.g., Tononi and Edelman, 1998; Singer, 2001; Koch et al., 2016; Nani et al., 2019). However, we would first need to gain a proper understanding of the underlying mechanisms of consciousness to get to grips with these problems. To start with, consciousness is most likely deeply rooted in the physiological processes of the nervous system. Although we are continually improving our understanding of local and interareal signaling in the brain, it still seems puzzling that electrochemical reactions at neurons and their synapses are probably responsible for consciousness. Why is it that something as subjective as one’s own pain is created by these electric currents and chemical reactions, whereas the very similar activities in other parts of our brain or outside our brain lead to either completely different feelings or to no obvious consciousness at all (at least not to you)? Understanding consciousness from a natural science perspective, including the underlying neuronal activities, holds promise for successfully manipulating our own consciousness maybe even without the coarse means that we use today, like taking an analgesic when we are in pain. One does not have to go as far as the phenomenologists and claim that studying consciousness should have priority because everything that matters to us is only accessible through our personal consciousness (Husserl, 1912-1929/1976). That consciousness is an obviously important matter for our scientific understanding becomes clear by merely looking at the fact that our experiences, including ones that we want to get rid of, such as pain or suffering, and others that we crave—joy or euphoria—are all taking place within consciousness. Thus, understanding human consciousness, including its presumed neuronal underpinnings, promises a wide range of beneficial applications.

The present article is concerned with one particular challenge in this endeavor to understand consciousness: the proper description of the principles of our subjectively felt consciousness itself. Like any object of sufficient investigation, there are certain characteristics that rule consciousness. When looking at one’s own consciousness—a method called “introspection”—we can note, for example, that a “stream of changing objects or contents” is typical of our personal consciousness (James, 1890). We have to face the challenge of describing these characteristics of consciousness in a more systematic way if we ever want to succeed with our natural science approach to consciousness. Without proper knowledge of what to explain, it is not possible to come up with an explanation. Consider an important example: the search for the neural correlates of consciousness (e.g., Tsuchiya et al., 2015; Koch et al., 2016). Empirical work on this topic in cognitive neuroscience needed an operationalization of consciousness that was compatible with available measures and typical experimental contexts. Specifically, these correlates were accessed by making contrasts between measures of brain activity occurring during conscious access to some information vs. during non-conscious processing of the same information (see Rees et al., 2002, for a review). Whether or not a stimulus was available to an observer’s conscious experience was quantified by their first-person report, and the contrast was made possible using paradigms with subliminal (masking, binocular rivalry) or preconscious (inattentional blindness) perception. It is certainly true that without a proper description of the principles and characteristics of what subjective consciousness would appear like if we were to find it, it is difficult to get the experimental set-ups right, in which we could hope to isolate the determining factors for conscious versus, for example, consciousness-independent processing. Note that there is also abundant evidence for the latter, for instance, in the form of non-conscious human processing (e.g., Marcel, 1983; Dehaene et al., 2006; Ansorge et al., 2014). However, we believe that hitherto the concept of consciousness used in the search for neural correlates is too narrow.

What we propose in the current article is to use research in empirical aesthetics, predominantly with art, for a fuller picture of the principles of consciousness in perception. Empirical aesthetics are mostly concerned with the understanding of aesthetic responses, aesthetic emotions, or beauty (*cf*. Leder et al., 2004; Leder and Nadal, 2014; Menninghaus et al., 2019), be it in art or nature (Fechner, 1876). Art perception can be so rich, full of surprises, and hitherto-untested experiences that many of us return to museums and galleries over and over again. It is as if the memory of art itself is not enough: To fully appreciate art, we need to experience art first-hand *via* our own perception. This is by no means restricted to the visual arts of galleries and museums. We can observe the same longing for repeated experiences when we finish a great novel, when we listen to our favorite music again and again, or when we go to theatres to see great motion pictures, a ballet, or a drama for a second or fifth time.

Therefore, in the present article, we focus on one particular area of consciousness where research on empirical aesthetics of art could be particularly helpful: perception. However, we hasten to add that perception is linked to other conscious functions such as memory (e.g., Hardcastle, 1995), imagination (e.g., Farah, 1985; Zeki, 1992), or emotions. For instance, many theories of emotions already include particular perceptual states as triggers or critical preconditions for the experience of feelings (e.g., James, 1884; Solms and Friston, 2018). These connections of perception to memory and emotion are certainly true of art perception, too (Leder et al., 2004; Pelowski et al., 2017c). Thus, although our focus is on perception, research on empirical aesthetics of art also sheds a light on other facets of consciousness to the degree that these facets overlap with principles characteristic of perception.

## Empirical Aesthetics of Art as a Way to Study Consciousness

As with any experience, experiencing art depends on consciousness. To understand this, consider a few examples. You feel lifted, energized, or are carried away by listening to your favorite music. You are so absorbed by a novel you are reading. You delay going to sleep and you have the feeling you lack the time to return to your novel and continue reading as much as you want to. When the light comes on in the cinema, it almost feels like you wake up and return from a dream. When you want to see how Velasquez painted the collar of a princess you get closer and can hardly believe how the paint is in fact made of brushstrokes of abstract patterns that only coalesce from a distance into the image of the tulle—a perceptual replenishing. What makes art experiences, and any other experience, wherever we are on the sociocultural gradient of what “art” could mean,^1^ conscious is that you can introspectively access its multiple qualities and, in fact, there is no alternative to introspection to access your first-hand experience.

In the present article, we suggest to use this dependence of art experience on consciousness for a better understanding of consciousness itself. In the following, we will develop our argument by first looking at a few principles of consciousness before we turn to examples of how art experiences could cast a light on the principles of conscious perception themselves.

### A Closer View of Conscious Perception: Attributions as a Core Topic

Introspectively, the diverse contents of our experiences come with one striking common characteristic: The bearer of the experience typically has a hunch about the origin of the contents of consciousness. In perception, for example, this has been emphasized by Helmholtz (1867), who argued that any perception has the quality of an “unconscious inference,” meaning the perceptual content has the quality of being about something in the surrounding world. When we look at a vase in front of us, for example, we experience the vase to be a physical object in a three-dimensional world. The vase occupies a particular location, it is of a specific material (e.g., glass), it has a particular color (blue), shape (cylindrical), and size. Typically, the possibility that these perceptual experiences do not originate in the outside world does not even cross our minds. Thus, unless in sophisticated or metaphorical extensions of its original meanings (e.g., as in “self-perception,” Bem, 1972, or in “unconscious perception,” Marcel, 1983), perception is literally defined as conscious content of a particular origin in the world surrounding us, including, of course, one’s own body, which is also part of the surrounding world. In addition, it is central to the origin of my perceived content that it is judged as “present,” situated in time at the same moment as the perception (rather than being felt as past like in a memory or as having never occurred like in imagination or dreaming). This has been more sharply worked out by Brentano (1874) as we will explain next.

The notion that, introspectively, conscious content (or once again the various “qualia” of our perceptions), is characterized by a hunch about their origins has been generalized to classes of experiences beyond perception. Brentano (1874) listed a number of different possible origins of our conscious content, such as dreaming, remembering, or imagining, that all differ from classical “perception” in that, to varying degrees, they point to us, humans, as the explicit origins of the present contents of our consciousness. “Remembering” can take on the form of having perceived something in the past and, thus, attributing the content of consciousness to both a past origin outside and also inside of our own mental sphere or consciousness—that is, a past origin as a perceptual object in the surrounding world and a present origin inside our memories. In contrast, dreams and imaginations lack this defining quality of necessarily having to have any resemblance to a past (perceptual) experience. When I imagine the stars falling to earth, I never had to have this perceptual experience in the past (and would very likely not even exist anymore to imagine this event if I had). The same is true of dreaming, but, in contrast to imaginations, dreams appear to originate more independently of our own plans, strategies, and will. Thus, introspectively, both dreams and imagination have an origin inside our own consciousness.

These different possible origins of conscious content bring up an important research question: How do we attribute conscious contents to their most likely origins? Consider the example of perception. There are at least two broad possible origins of conscious content that have sometimes been labeled as the “distal” versus the “proximal stimulus.” For example, think of the vase that we see and the pattern of reflected light that reaches our retinae (i.e., the light-sensitive areas of the human eye) that corresponds to the vase; or the sonata that we hear and the waves of compressed air created by the sonata that reach our inner ears. Introspectively, we seem to attribute our perceptual contents swiftly and with certainty to the distal object. Here, Heider (1921/1959) asked how it comes that we attribute our perceptions to the distal stimulus, such as the vase, rather than to the proximal stimulus—in this case, the pattern of light energies that impinges on our retinae. This question is justified, as without the proximal light pattern as a stimulus, there would be no perception of the distal objects surrounding us either.

In response to the question above, Heider argued that we attribute conscious content to that part of the physical surrounding world that seems to rule the other part, namely the distal stimuli that seem to rule the appearance of the proximal stimuli. With respect to this distinction, Heider coined the terms “objects” for the ruling (distal) physical stimuli (e.g., a vase) and “media” for the ruled (proximal) physical stimuli (e.g., the light patterns on the retinae). Heider’s considerations concern the same process that Helmholtz (1867) labeled the “unconscious inference.” Thus, there is typically not much conscious musing about the possible origins of our perceptions. Typically, attributions work swiftly and leave little space for doubts, so that perceived objects in the surrounding world appear as what they are.

This pivotal phenomenon is discussed in great detail in the field of philosophy of mind/consciousness, where it is referred to as “transparency of experience” (e.g., Harman, 1990). The claim is that when we try to introspect our own (e.g., visual) experiences, we “look through” our sensory experiences, and we seem to be directly aware of the external objects rather than of the properties of the experiences themselves. From a semantic perspective, this implies that the meaning of the external object is immediately present to our conscious experience in the moment of perceiving or thinking about the object. We cannot stress enough how central this feature of attribution—assigning an origin to a consciousness content—is for perception. This process of attribution is not only evident in the perceived object itself, it also contributes to the felt difference between just any perception—being about a present object—and other qualities of consciousness (e.g., memories). Thus, through understanding the principles of such attributions, we might find ourselves in a position to study an important characteristic of any conscious perception.

A question related to that asked by Heider (1921/1959) concerns the attribution of the perceptual content to the surroundings or to the distal objects rather than to our own actions. During perception, humans carry out actions, such as directing their gazes to different areas of an image or converging versus diverging the eyes to focus on a particular focal plane of their three-dimensional environment (e.g., Lisberger et al., 1987; Henderson, 2003). What is true of the eyes is true of the whole body: humans turn their heads and move around during perception and, thereby, collect important information about the invariant properties of their surroundings (Gibson, 1966). In general, actions have the capacity to change the status of objects, including their exact perceptual appearance. Think of touching a painting with your fingers, leaving a fingerprint on its surface, or grasping a vase to turn it so that its perspective changes. Although actions are involved in perception, as all these examples illustrate, introspectively, we humans attribute the resulting conscious content often entirely to the objects rather than to our own actions, seemingly assigning our own actions in the service of perception a similar status as the proximal stimuli (Engel et al., 2013): They are “media,” to follow Heider. Equipped with this brief look at some basic characteristics of consciousness in general and of perception in particular, we now turn to examples from art experience to elucidate how the empirical study of art may mark the way to a deeper understanding of consciousness.

### Examples of Empirical Aesthetics of Art as a Means to Study Attributions in Conscious Perception

According to some modern approaches and developments in art theory and practice, any action or its outcomes can become an example of art (e.g., Benjamin, 1969; Danto, 1974; Virilio and Lotringer, 2002; Beuys, 2007). However, this definition leaves open how art relates to aesthetics, and, at this point, our simple proposition is that objects of art are accessible through individual perception and that introspective experiences attributed to a work or an object as “art,” thus, form an important class of aesthetics (*cf*. Zeki, 1999; Leder et al., 2004; Jacobsen, 2006; Shimamura and Palmer, 2012; Palmer et al., 2013). Originally, “aesthetic” (Greek αἰσθητικός, or “aisthetikos”) meant “sensitive” or “sentient,” and, in the underpinnings of to what individuals were attuning, referring roughly to the same types of sensory or perceptual fundamentals as the concept of qualia (Dennett, 1988), but at the same time emphasizing their inherent value. The latter was further brought to the foreground by Baumgarten (1750/1988) who aimed to establish a research discipline seeking to explain beauty as the common denominator of qualia-inherent value or worth, nowadays often measured by preferences or judgments (e.g., Kuchinke et al., 2009; Ishizu and Zeki, 2011; Muth and Carbon, 2013; Vartanian et al., 2013; Miller and Hübner, 2020; Spee et al., 2022). The concept of the aesthetic has undergone further shifts of meaning in the Kant (1970), analytic, and modern views of aesthetics (e.g., Shusterman, 1987; Mattick, 2003), but, here, it suffices to conclude that as long as an inner connection to qualia is granted, empirical investigations into aesthetic experiences mark a promising path to the study of conscious perception.

At this point, two remarks are necessary in order to prevent any misunderstandings. First, the reader should note that we do not claim that works or objects of art are the only origins of aesthetic experiences. There are alternative origins of aesthetic experiences in the perception of natural objects, not just in the perception of pieces of art (e.g., Augustin et al., 2012). The different aesthetic experiences created by art and by natural objects might be related. However, the quality and degree to which these different aesthetic experiences are related are largely beyond the scope of the present article. Second, the reader should note that others might use the word “aesthetics” with a different meaning than we do, for example, to refer to concepts that are independent of conscious experiences (e.g., Purchase, 2002). However, in the current article, whenever we speak of “aesthetics” we use this term as a short-hand and synonym for “aesthetic experiences.” In the following paragraphs, we discuss some instructive examples of how art aesthetics could inform consciousness research.

#### Duchamp’s *Fountain*

**Figure 1:**
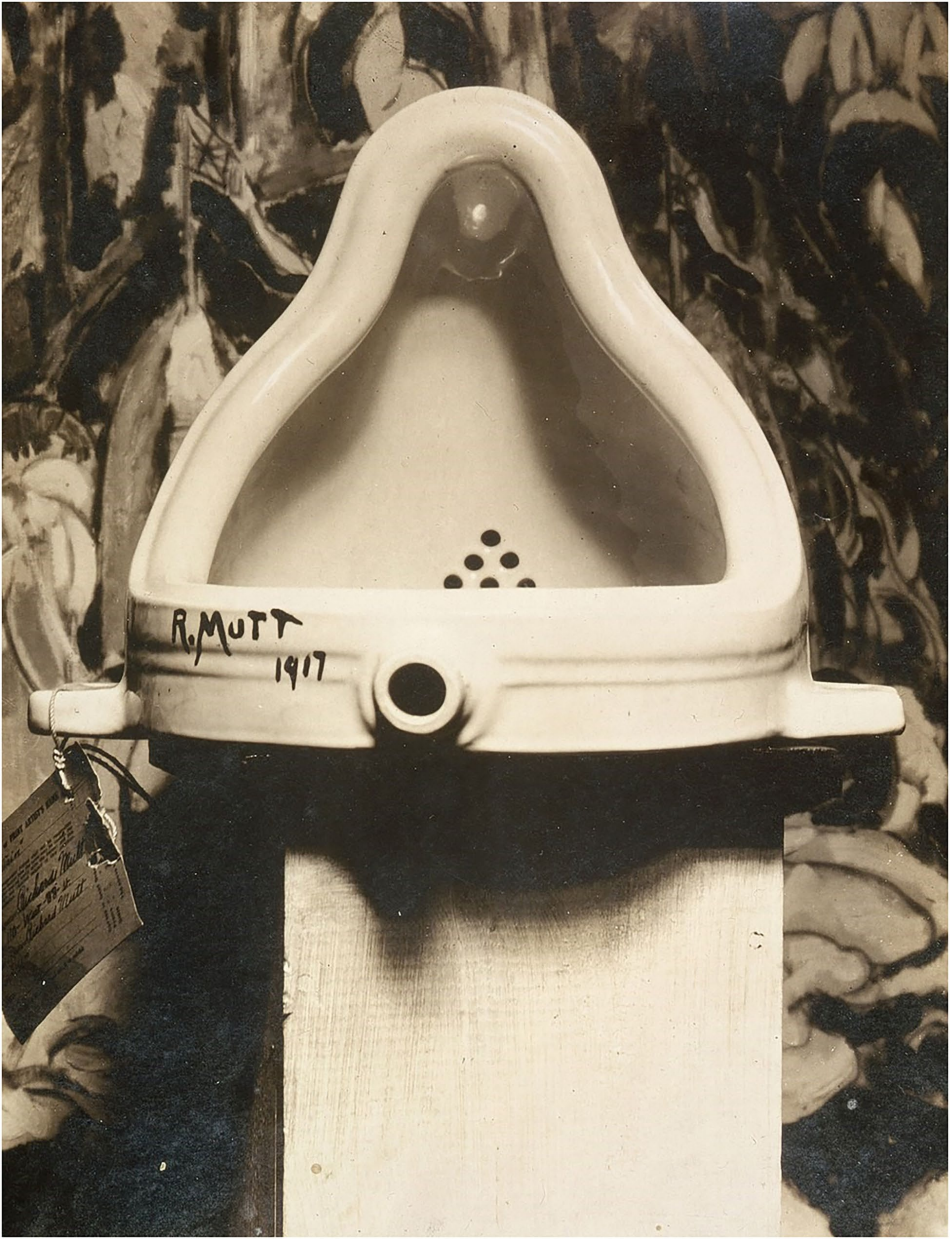
*Fountain* by Marcel Duchamp as photographed by Stieglitz (2017). Reproduced with permission.

We start our examples with Duchamp’s *Fountain*. In 1917, Marcel Duchamp presented a standard male urinal, rotated by 90°, as a work of art of the class of the so-called ready-mades in the *Gallery 291* in New York. Not many have seen the original, but it was photographed by Alfred Stieglitz (see Figure 1) and, thus, got enough publicity to trigger a discussion about art with repercussions until today. *Fountain* is interesting, as it is not readily apparent why this object could have triggered an aesthetic art experience. In fact, many viewers might not subscribe to the view that *Fountain* is a piece of art and, thus, would deny that it triggers an aesthetic experience. However, from the perspective of consciousness research, it is clear that *Fountain* must carry the critical ingredients for an aesthetic experience if at least some of us experienced it aesthetically. In other words, a closer scrutiny of the critical determining factors of the exposition of *Fountain* promises insights into the operation of consciousness itself. What *Fountain* shows is that using an object (here, a urinal) outside of its typical context (e.g., neither in a public restroom, nor at a junkyard, nor in a sanitary warehouse) and/or in a novel context (here, in an art gallery) could have stripped the object of some of its original affordances (but see Goffman, 1974)—that is, its perceived typical means of usage (Gibson, 1966; here, for urination)—and, at the same time, allows the object (here, the urinal) to take on the role of a medium referring to a novel distal object of art originating more in our imagination and less in only our perception.

Relatedly, the perceiver attributes the urinal’s placement of the object to the willed act of the artist, an attribution that is a major reason for the aesthetics of *Fountain*. Stripping an everyday object of its obvious functions and placing it in an unusual configuration and place would then be conditions for this important inference. These deliberations point to the fact that one may consider willed designs or actions as necessary preconditions for the perception of an object as a work of art. For such an attribution in the aesthetics of works of art to succeed, it is not necessary that the underlying assumptions of the perceiver are (entirely) true. In other words, humans might err in assuming a work of art as an outcome of an act of free will or of deliberate choice on the side of the creator, and they could still use this criterion for their attribution of aesthetic experiences to a work of art *(cf*. Gell, 1998).

From a theoretical perspective, these observations are not surprising. The more general issue raised here concerns the question of how humans perceive material artifacts and how they attribute meaning to them. Though originally not meant to elucidate conscious perception and despite Gibson’s (1975) skepticism regarding the prospects of empirical aesthetics, we suggest looking at questions pertaining to the conscious perception of the material world through the lens of affordances (Gibson, 1986). According to Gibson’s (1986) classic definition, affordances are possibilities for action offered to a cognitive system during perception of an object in the environment; they are an opportunity for action and not primarily a property of the object. An object “invites” the human perceiver to interact with it according to the perceiver’s abilities. In this context, Gibson also introduces the notion of a niche as actively transformed and tailored places that fit the needs of a perceiver. As shown by Rietveld and Kiverstein (2014), Gibson’s concept of a niche implies that the very same material environment can offer many ways of using, interpreting, and interacting with it. Thus, it may be more useful to speak of constraints rather than of affordances (*cf*. Norman, 1999). In the case of human ecological niches, we can see a wide diversity of sociocultural practices: regular and socially agreed patterns of behavior (and corresponding assignments of meaning), oftentimes of a normative character (e.g., behavioral practices a human feels he or she is obliged to follow). They are expressed as skills (understood as well-practiced perceptual, cognitive, and behavioral capabilities), typically acquired as an adaptation to the general norms expressed in these sociocultural practices themselves. Hence, the diversity of possible affordances is constrained by these sociocultural norms.

In other words, we are “educated” in our attention and in our ways of perceiving affordances relevant for our needs and (intended) action possibilities as they are offered by a particular object of perception. Ingold (2013) suggests a relationship of correspondence between the structures of the material environment, the possible behavioral interaction patterns it offers, the abilities (and constraints) of the perceiver, and how well the concrete perceiver’s behaviors fit into the socio-material environment. An affordance would, thus, be a socially agreed correspondence between an object (such as a urinal) and its “appropriate” use.

The important point, though, is that an affordance is both subjective and objective. As is pointed out by Gibson, an affordance “is equally a fact of the environment and a fact of behavior. It is both physical and psychical, yet neither. An affordance points both ways, to the environment and to the observer” (Gibson, 1986, p. 129). Why is this important? From an affordance perspective, there is no “objective” or “correct” interpretation or usage. Although most artifacts (including objects of art) are designed with a specific function or meaning in mind, they offer a rich “landscape of affordances” (Rietveld and Kiverstein, 2014); they act as an interface inviting the perceiver to engage in various forms of interpretation and behaviors. Hence, the perceiver is always confronted with a bundle of affordances, however—in most cases—chooses the reaction according to his/her (socially) learned skills (in the sense of being constraints).

Whether a particular affordance becomes relevant, does not only depend on learned skills, but also on the concrete situation or niche in which one finds him−/herself as well as on the current concerns and interests of the perceiver. In such situations, it is possible that we engage with a novel or even sometimes unintended affordance leading to interpretations or attributions of meaning beyond the intended and/or socially agreed use.

The analysis of *Fountain* makes clear that the perception of objects of art and their associated attributions could differ between individual humans and that aesthetically ambiguous objects of art in this way provide an interesting approach to the study of consciousness. Aesthetically ambiguous objects of art could be used to study, for example, brain activities correlated with the different attributions created in the course of conscious perception in between-participants designs—with one group of participants prompted to have, and a different group more likely not have, an aesthetic experience in response to the same object (*cf*. Kirk et al., 2009; Huang et al., 2011; for a review, see Pelowski et al., 2017b). Alternatively, one could conduct a within-participant comparison of attributions created before and after learning about the defining context of an artwork. In this way, such studies would allow to investigate the process of attributions associated with different conscious perceptions in detail but without confounding visual differences between the stimulus materials. Studies comparing judgments or processing of experts and novices (here, of art perception) aim in this direction, but so far too little research is devoted to the corresponding introspective data that would accompany the rating and processing differences between these groups (e.g., Weichselbaum et al., 2018; Leder et al., 2019; Pelowski et al., 2020). With the current framework, we hope to inspire the development of research designs that fit this purpose, for instance, asking participants directly about their attributions to deliberate versus less deliberate acts of a creator or asking participants about their assumptions regarding the intended purposes of the acts manifested in (ambiguous) works of art.

#### Duchamp’s *Nude Descending a Staircase*

**Figure 2:**
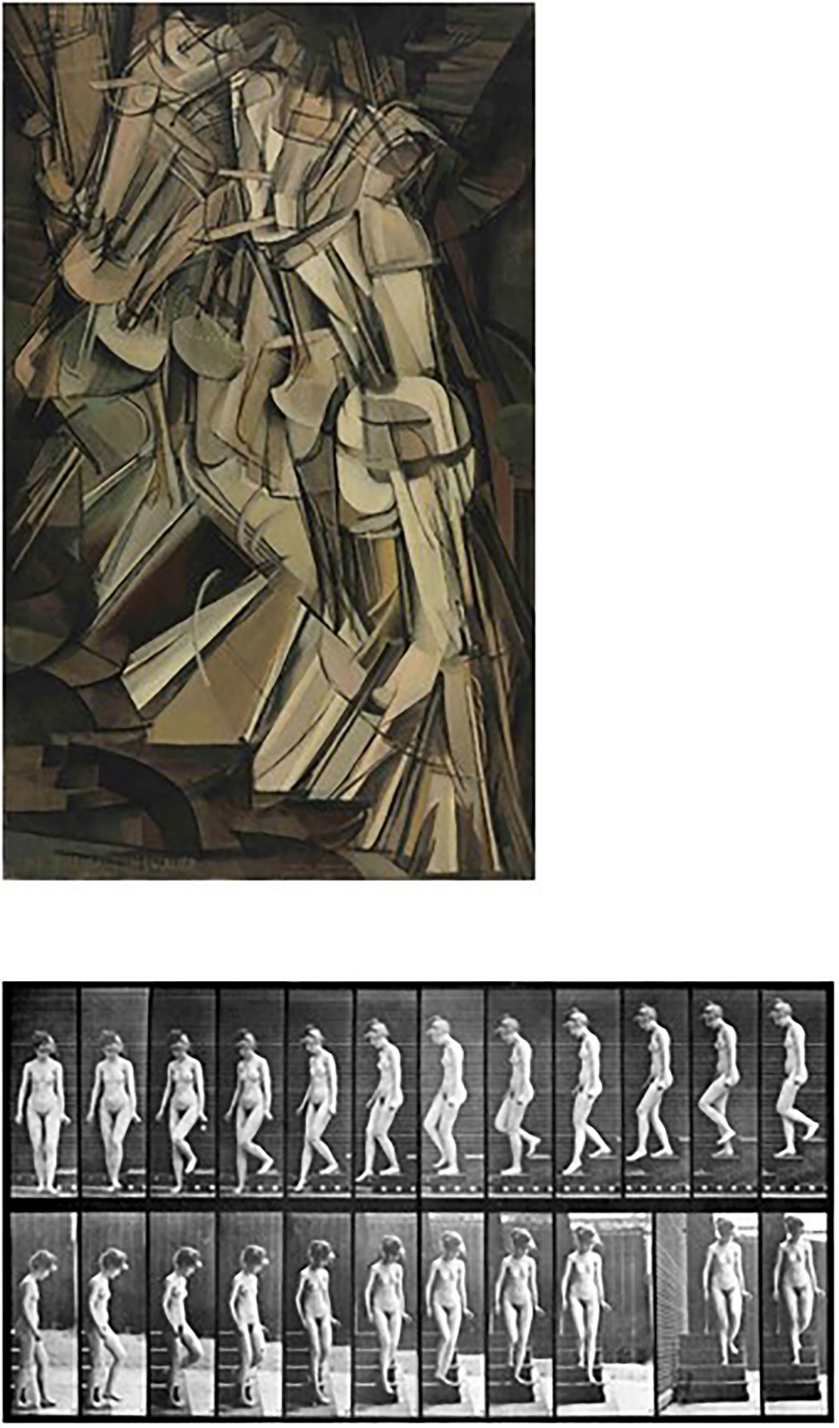
Top: *Nude descending a staircase No. 2* by Marcel Duchamp (1912). Bottom: *Woman walking downstairs* by Muybridge (1887). Reproduced with permission.

In 1912, Duchamp painted the outlines and relatively monochromatic surfaces of the effectors of an abstract body as if simultaneously present in different phases of its movement (a descent) on a spiral staircase (see Figure 2). This work of art uses an existing medium—a static painting—to convey an object in motion as its content and, thus, extends or experiments with extending the defining limits for what types of objects could be successfully represented by this particular medium. For this piece of art to be “understood” by the perceiver, it is necessary that viewers attribute the simultaneously presented body images to the successive phases of the body’s motion, an attribution facilitated by the analytical depiction of animated motion in series of photographs of the type first shot by Muybridge in 1878 (see Figure 2, for an example from 1887). In other words, Duchamp endowed the painting as a medium with a characteristic inspired by a different medium—the photograph (*cf*. Ramachandran and Hirstein, 1999).

With respect to consciousness research, it would be possible to more systematically use these kinds of insights into potential differences and similarities between media. For example, different media (e.g., paintings versus photography) could be rendered similarly with respect to differences in their typical respective characteristics (e.g., their color palettes, depth renderings, representations across time; for the latter, see the example above in Figure 2) to study which of these differences are decisive for the attribution of the perceptual content to a particular medium rather than to a specific object. Only then could it also be investigated if and how media differences show up in or contribute to the contents of perception in general and to the perception of objects in particular (*cf*. Quiroga et al., 2009).

#### Demand’s *Control Room*

**Figure 3:**
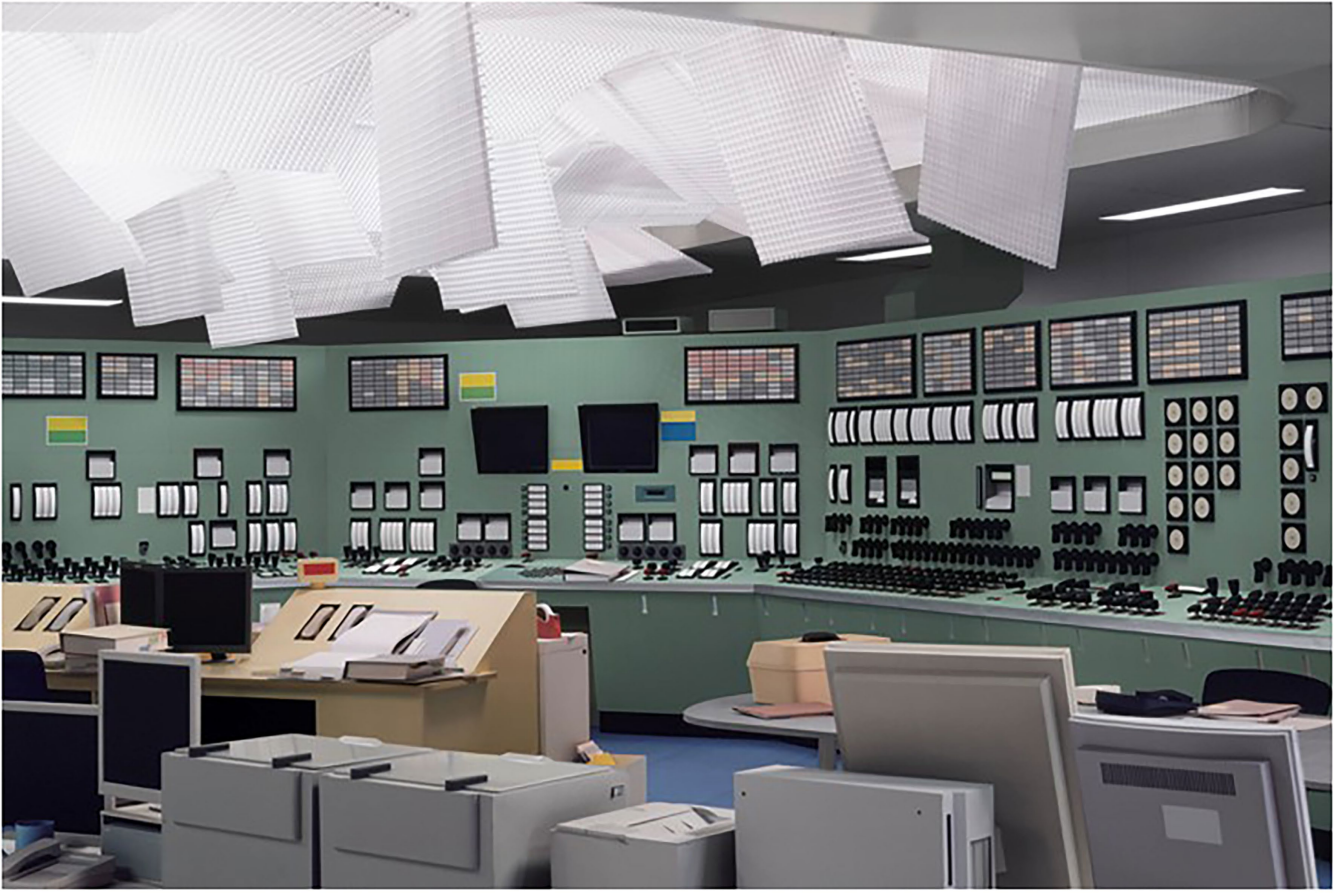
Thomas Demand’s *Kontrollraum/Control Room* (2011), C-Print/Diasec, 200 × 300 cm © VG Bild-Kunst, Bonn. Courtesy Sprüth Magers Berlin London. Reproduced with permission.

In 2011, Thomas Demand created a three-dimensional paper model of a control room from folded, cut, and glued paper and took a photograph of this model using a perspective from which the model appeared as a real control room (see Figure 3). This is just one example of many conceptually similar works of art by the same artist. Those photographs address the relation between medium-specific limitations and the appearance of the represented objects, here, the limitation of photographic stills taken from a single perspective that restricts access to alternative perspectives that would inform about the veridical object of perception. In addition, those objects can trigger active search and processing strategies to confirm the suspected artificiality of the photographic contents: Once the viewer is informed about the artificial nature of the photographed scene either by stumbling across slight deviations from real objects in the process of a visual scrutinization of the photograph or simply by prior information—the image actually depicts the control room of the Fukushima power plant but without signs or numbers, raising a sense of inconsistency between perception and memory—it is possible for the perceiver to actively search for additional evidence for the artificial nature of the perceived object (*cf*. Huang et al., 2011).

In consciousness research, such pieces of art could be used for studies on the impact of attributions of perception to “real” versus “artificial” objects under visually similar conditions, once before and once after instructions informing about the true nature of the objects as mere paper imitations of physical objects. This type of research has the potential to reveal differences between consciousness-dependent processing of real and artificially simulated objects free of visually confounding differences.

#### Stockhausen’s Spherical Concert Hall

**Figure 4:**
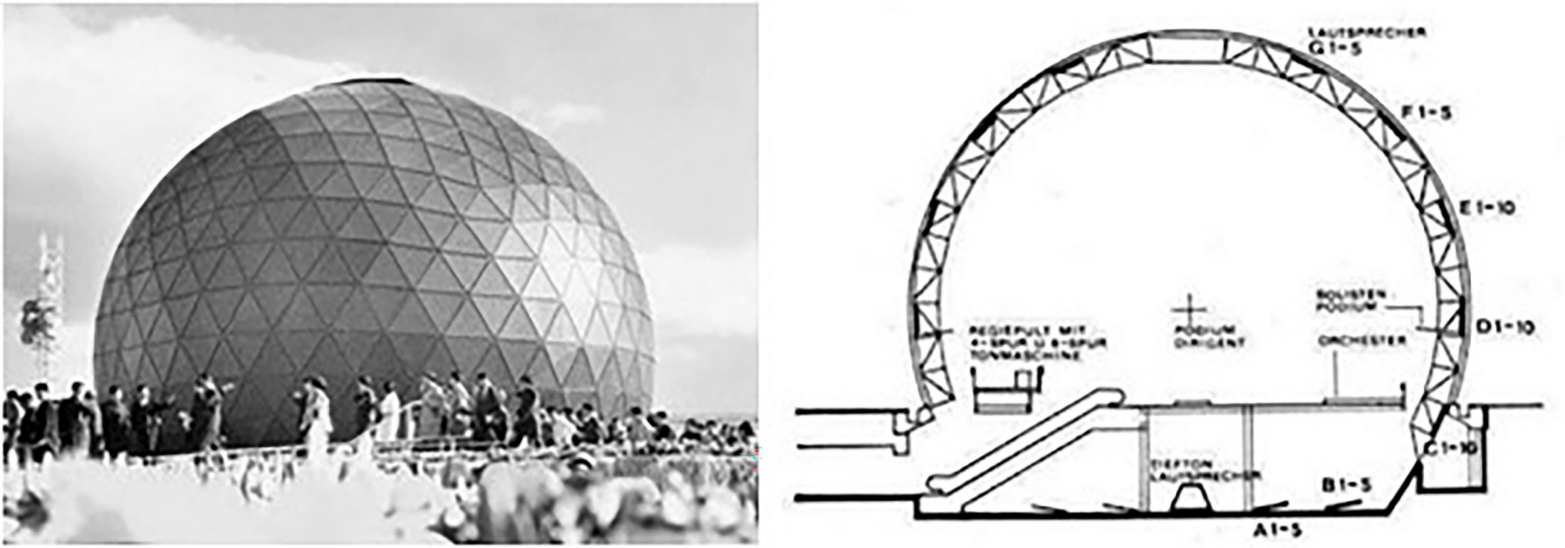
Left: Stockhausen’s spherical concert hall created for the World’s fair Expo ‘70 (1970) viewed from outside (Stockhausen, 1970a,b). © Karlheinz Stockhausen. Right: Lateral cut of the spherical concert hall. © Stockhausen-Stiftung für Musik, Kürten (http://www.karlheinzstockhausen.org). Images reproduced with permission.

In 1970, the composer Karl-Heinz Stockhausen designed a spherical concert hall for the Expo in Osaka, Japan, allowing him to play music from every direction (see Figure 4). At this time of his career, Stockhausen was occupied with the systematic inclusion of space into the composition of music (e.g., Miller, 2009). Space and direction had been important compositional and performative characteristics of music long before (e.g., Howard et al., 2009), but Stockhausen took those spatial characteristics of the medium to their rarely (or, actually, until this point probably never) used extreme.

Regarding research on consciousness, this raises the question of how the full or more exhaustive use of medium characteristics can alter object perception compared to the more conventional use of only a restricted range of the same characteristics. For example, the exhaustive range of medium characteristics could provide a space of reflection upon the perceptual content that is not provided by its more constrained implementations (*cf*. Arendt, 1958). Such questions could be studied across different characteristics (or dimensions as they would more typically be called with respect to the sensory processing of humans), such as space and time (the latter as actually also done by Stockhausen), both with more passive recipients and with actively roaming observers, and even across different sensory modalities (e.g., sound and vision). Real-time augmenting and immersive technologies open a gate to tackle these novel possibilities, including the manipulation of sensory-motor coupling (*cf*. Davies and Harrison, 1996; Nagel et al., 2005).

## Why Research on Empirical Aesthetics of Art Rather Than of Perception Under Natural, More Ecological Conditions?

**Figure 5:**
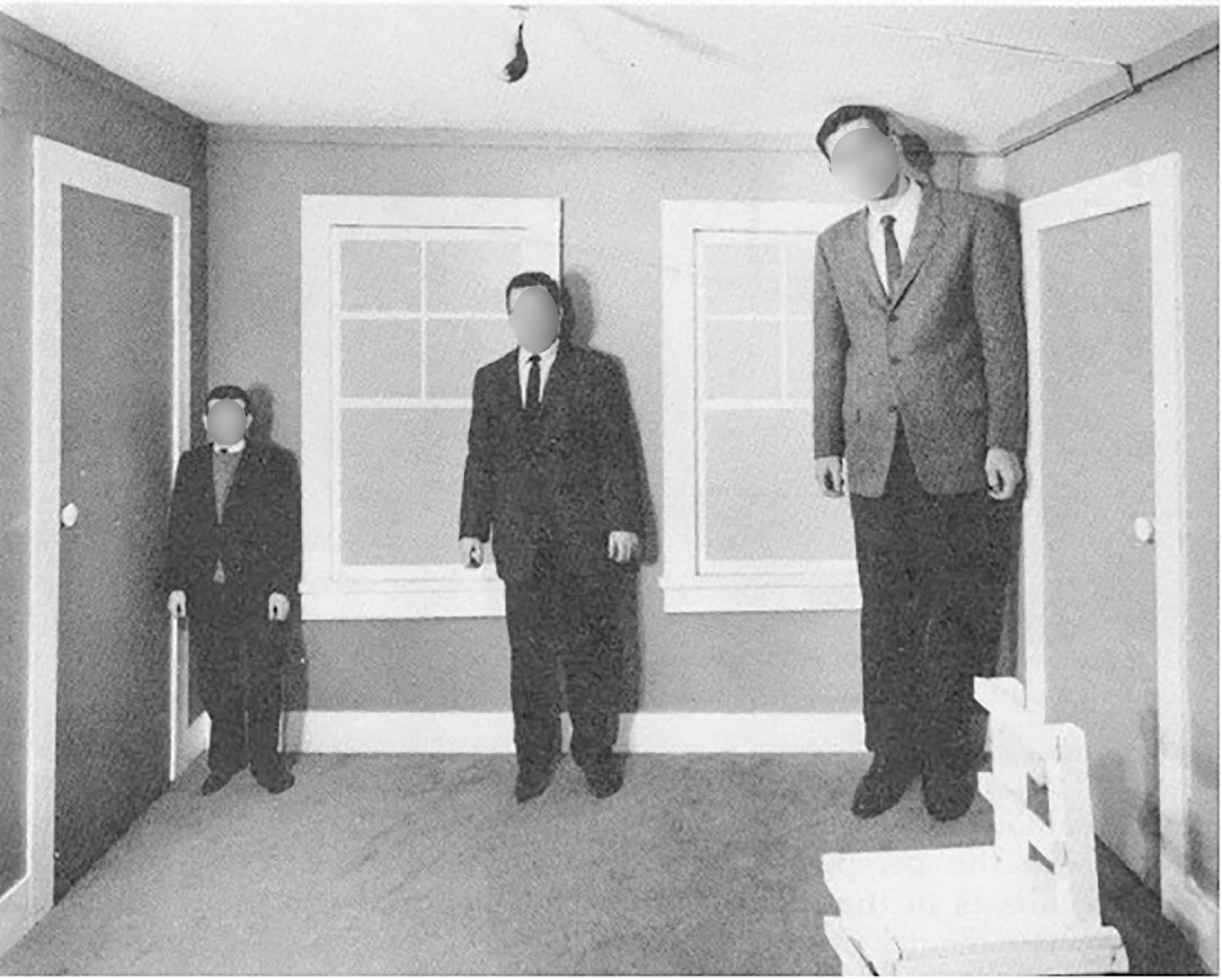
Photograph of an Ames’ room size illusion. The viewing distance of the right corner is closer than the viewing distance of the left corner, but from a particular perspective the correspondingly tilted edges between floor and background wall and between ceiling and background wall appear parallel so that about equally sized men located alongside the background wall from the left to the right corner seem to vary in size. Retrieved from http://www.anopticalillusion.com/2012/07/vintage-ames-room-illusion/. Reproduced with permission.

At this point, we need to answer a question that has been posed to advocates of highly controlled empirical research on perception in general. Critiques have questioned if it is sensible to study perception under the highly artificial conditions of the laboratory, as those conditions differ from perception under more ecological conditions in many relevant ways (Gibson, 1966, 1979). For example, Gibson argued that many perceptual effects, for instance, visual illusions such as the size distortions in Ames’ rooms (see Figure 5), would not be observed if participants were allowed to freely move their heads or bodies around in 3D space rather than having to view a scene from a single perspective (for corresponding evidence, see Gehringer and Engel, 1986).

**Figure 6:**
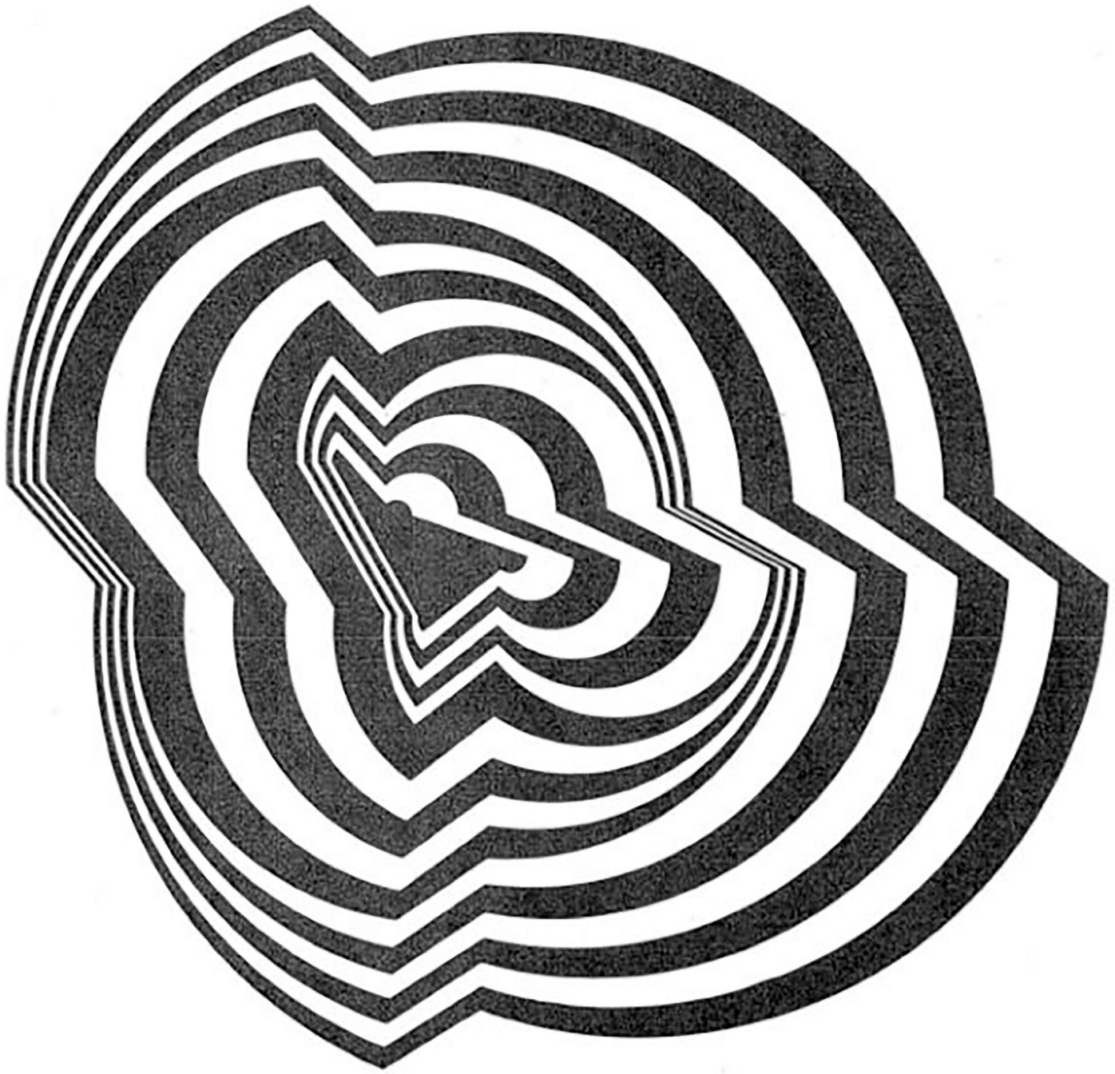
*Disfigured Circle* by Bridget Riley depicts depth from aligned angles (Shapley and Maertens, 2008). © Bridget Riley. Reproduced with permission.

In our view, the same criticism could rightfully be directed against our suggested approach of using empirical aesthetics of art to inform research on consciousness. The reason is that if highly controlled laboratory research is already different from perception under the most common, ecological real-world conditions (Pelowski et al., 2017a), art does deviate from these conditions even more. In our view, it is no surprise and rather typical that controlled laboratory research and aesthetic art experiences have both addressed perceptual illusions (e.g., Troncoso et al., 2009; Martinez-Conde and Macknik, 2010; Bacci and Melcher, 2011) that are maybe not very common under real-world perceptual conditions (Gibson, 1966, 1979). Think of *Op Art* as an example. In *Op Art*, artists created images seeking to elicit illusionary perceptions (see Figure 6).

This inclination toward illusions is typical for art itself (Shepard, 1990), as art in general almost always directly or indirectly violates or at least “addresses” (i.e., problematizes, calls into question) the exact relations between object, medium, and perception. The reason is that art typically relies on media over and above the most basic natural media, such as light or sound. Importantly, justifying the skepticism regarding the ecological validity of the conditions of art perception, art almost always uses object-medium relations deviating from “natural conditions” and often even from previously used “artificial conditions” (e.g., controlled laboratory conditions). Paintings, photography, cinematography, electromagnetic tape recordings, musical instruments, monitors, amplifiers, digital storing devices, tonal scales, languages—all these media change preexisting, “basic” natural media. Often they limit the characteristics of natural media (e.g., a photograph only covers part of the natural contrast range for which the eye is sensitive), or they can alter the ways in which objects and actions relate (e.g., natural 3D spatial coordinates behave differently relative to observer’s self-motion than “artificial” 2D coordinates of images of objects); sometimes they extend them (e.g., synthesized sounds can be added to the repertoire of naturally occurring sounds), and sometimes they cross-cut the boundaries between natural media (e.g., if sounds are visualized by color devices).

Superficially, it might seem as if some performing arts (e.g., acting) or sculptural arts (e.g., wood carving) would be exempted from this artificiality or lack of ecology, as they do not seem to “distort” or “compromise” media over and above the natural ones. However, this view does not acknowledge that actions in performing arts are media *per se*, that the used materials in sculpturing (e.g., stone, wood, glass, plastic) also endow objects with important characteristics (e.g., possible coloration techniques, different changes across extended times, perspectives, etc.) hitherto not owned by kin objects as they appear naturally, and that both performances and sculpturing change natural media characteristics already simply by increasing the range of objects that can possibly be conveyed. On top of this, artists even use artificial media in unconventional ways, trying to increase the scope of objects or characteristics covered by a medium. Our examples above were all illustrations of these less conventional relations between objects, media, and perception in the aesthetics of art.

In addition, that art provides alternatives to the natural or art-predating relationships between objects and media is also a commonality evident in very different and even opposing stylistic approaches to art that have been advocated by artists and in art theories. To understand this, just think of one major opposing difference between stylistic approaches: should art strive to imitate reality, and if so, how could art ever fulfill this purpose (e.g., Adorno, 1970; Benjamin, 1977a,b; Gombrich, 1960; for examples from cinematography, see Bazin, 1962/1976; Kracauer, 1960/1985)? This could not possibly be a question or a task for artists if art’s capacity for imitation were somehow certain or secured. This can only be a matter of striving and debate if art could noticeably alter these ecological or natural relationships between objects and media.

However, despite this seeming lack of ecological validity, we see great value in the empirical aesthetics of art as an approach to carry out research on consciousness in perception. Due to the inherent deviation from natural object-media relations, art often appears to be inventive or unconventional and “uninterested” or without obvious purpose beyond the creation of an aesthetic experience itself (*cf*. Schopenhauer, 1859/2009).

This in turn is exactly the reason that art is ideally suited to study human attributions in conscious perception, in comparison with protocols dedicated solely to the most common and conventional perceptual abilities observed under ecological conditions. Only by inclusion of the less conventional media-object relations of art can we be sure to exhaustively cover perception and to put our hypotheses to ever new tests rather than to inadvertently restrain our research and undermine the potential falsification of our conclusions. This means that by the very fact that ecological conditions are limited relative to the arts in varying and manipulating media and media-object relations, sticking to the ecological conditions in our investigations would carry the risk of overlooking important exceptions from our preconceptions about consciousness and perception. Having said this, however, we want to add that the ecological perspective on perception carries important lessons for an empirical aesthetics approach to consciousness. For example, the ecological perspective on perception emphasized the temporally extended nature of the perceptual act that leads to important distinctions such as reversibility versus irreversibility of temporally unfolding perceptual events. We therefore believe that research on empirical aesthetics of art, whether or not in the service of understanding consciousness, will definitely profit from including these lessons. For instance, the temporal structure of the unfolding perceptual events should not only be reflected upon when studying temporally extended works of art such as music, dancing, or cinematography. This perspective should also be taken to heart for the study of the more static forms of art, such as photography and painting, which observers still explore in a temporally extended manner.

A related advantage of using empirical aesthetics of art to study consciousness lies in the higher potential to bring to the foreground of conscious reflection the processes of attribution occurring during perception. In ecological or conventional conditions, those processes occur automatically and swiftly as implied by the label of “unconscious inference.” However, the creation of novel media, the unconventional usage of existing media, the novel media-object relations, and the endowment of existing media with novel characteristics in art objects all violate existing expectations (*cf*. van de Cruys and Wagemans, 2011) and, thus, have a good chance to be registered and noticed by the perceiver. This is suggested by the fact that expectancy violations capture attention and can, thus, facilitate perception of the expectancy-violating event (*cf*. Horstmann, 2002, 2005; Itti and Baldi, 2009) and by the information value carried by the differences between our predictions (or expectations) and the current status quo (*cf*. Friston and Kiebel, 2009; Clark, 2013). To the degree that introspective registering of an event is a precondition for its verbal report (see below), those characteristics of art aesthetics would, thus, be beneficial for the research aim of getting a fuller description of conscious perception. This brings us to the final point of the present manuscript, a more elaborate description of the method.

## Methodological Considerations: How to Get and Use Introspective Data

We have already explained that the description of consciousness requires introspectively looking at one’s own personal mental content from a first-person perspective. To date, there is no alternative way to access this conscious content. This also means that if we want to get insights into the conscious content of another person, we need to ask them about their introspections (*cf*. Ericsson and Simon, 1980). Thus, we will also complement the current suggested approach to consciousness research by a more elaborated (but still brief) look at the methods that could be used. In this context, methods from neurophenomenology, first proposed by Varela (1996), could be applied. One of the objectives of the neurophenomenological approach to the study of consciousness is to bridge the first-person/third-person gap, that is, to reconcile and integrate data from lived (first-person) experience and (third-person) data from neuroscientific experiments (e.g., EEG or fMRI studies). As Olivares et al. (2015) suggest, this approach could be complemented with second-person methodologies in which a trained interviewer acts as a mediator, supporting and guiding the subject in exploring and describing his/her experiences in a structured and professional manner in order to reduce subjective bias.

Basically, we recommend a three-pronged multi-methodological approach. First, we suggest that researchers reflect upon the possible perceptual effects of art-works on consciousness, just as we did in the examples above. This part of the method would allow first hypotheses about how exactly conscious perception unfolds in art aesthetics. When doing this, we should systematically vary the determining factors to see whether they stand the test of our perception (*cf*. Mill, 1889). For example, our reflections upon *Fountain* have led us to the hypothesis that an unconventional context of an object could change the aesthetic perception of an object as a piece of art. In a next step, we could personally search for other such examples, for instance, other “ready-mades” or other contexts that should work in a similar way on the same objects, for a larger sample of objects and contexts that could be used to put the same hypothesis to the test by our own introspection. Here, we could also artificially construct novel object-context combinations that hitherto do not exist as art works to test for the generality of the assumed principle (e.g., Markey et al., 2019).

Secondly, we should aim at testing the emerging hypothesis with a set of suitable possible objects of interrogation with a sample of different observers. Here, we could conceive of different groups, such as experts versus novices, persons of different genders, ages, socioeconomic status, or ethnicity. We could do this as in so-called theoretical sampling, until the qualitative interviews with these persons do not deliver (much) more novel insights (Glaser and Strauss, 1967; Strauss and Corbin, 1990). Interviews should proceed in two different phases: gathering unguided comments and then providing specific hypotheses to be discussed by the observer. In the first phase, we can present each selected object and ask the observer what they perceive, allowing them to include any prior experiences with a particular object of art, which may function as an ice-breaker to facilitate the flow of the interview. We should explain that in order to better understand the processes of the conscious perception of objects of art, we invite the interviewee to express whatever thoughts occur to them in the course of the perception of the object, at any time during its presentation. If we present an extended piece of art, such as a sonata or a (videotaped) ballet, we then would pause the presentation until the comment has been concluded. Repeated presentations of an artwork can be offered if desired by the interviewee. In this way, we will be able to potentially collect precious additional data about how our selected objects are perceived. The data might lead to conclusions that deviate from our original hypothesis, and generate new hypotheses for further interviews. The second phase of the interviews involves presenting our participants with our specific hypotheses and allowing them to ask clarifying questions if needed. They are then invited to provide their opinion: do they share our view or disagree, complement or deny our hypothesis? During the interview, we should also inquire specifically about any media and object differences between the situation we seek to understand and the presentation of the objects necessary for a full understanding of the content of the interview. For example, we could point out that we have a hypothesis of how *Fountain* is perceived in an art gallery and that this specific context is possibly unclear in the photograph used. For any answers of the interviewee, it should be made clear what object and medium she or he talks about (e.g., a ballet or a video-recording of the same ballet). All data from the interview should be analyzed with the typical methodological repertoire of grounded theory.

Third, once the analyses of the contents of the qualitative interviews have been concluded, we can develop a questionnaire in which we condense the major insights won during the interviews. This questionnaire could be used in an economical way to discern between participants with different attribution styles regarding art objects. They can then be invited to participate in further studies into systematic inter-individual differences in the conscious perception of these objects using experimental research designs adhering more closely to the repertoire of the natural sciences, such as eye-tracking or brain measures. In this way, our insights from research in empirical aesthetics could be used to study open questions surrounding consciousness, including the neuronal correlates of conscious perception. It is also important to ask participants about their perceptions following such investigations, again to confirm important assumptions that might otherwise be in doubt.

This general approach could also be used in the inquiry of the artist as the creator of intentional conscious reflections on art (*cf*. Augustin et al., 2008) or to study the transition from unconscious to conscious effects in aesthetics (*cf*. Leder, 2013; Leder et al., 2013; Belke et al., 2015) that can occur during an interview itself, or over the time course of any study.

The approach presented here, which was illustrated by case studies, must of course also be discussed critically. The conceptualization of consciousness that we examined is a concept we defined ourselves; other ideas of consciousness are, of course, conceivable. In addition, the examples given in individual cases give little insight into how exactly the processes of becoming aware work, but rather clarify how the transition from different levels of awareness can be examined with the help of art perception.

Another challenge would be to translate the features of the examples we discussed, as compelling as they might be, into testable experimental designs, in which the limitation of single case studies is overcome. This often requires a careful extraction of the aesthetic features and their multiplication for the necessary number of objects or trials for repeated measurements, and to assure its effect across different observers to confirm generalizable conclusions. As such, the present paper aims to demonstrate the strengths of art as a tool to understand consciousness, and a convincing, empirical, generalizable understanding of the components involved is certainly a promising, but also exciting task for future research.

## Discussion of Time and Creativity

Before we conclude our argument, we briefly want to discuss the role of time. So far, our sketch of perception did not acknowledge the complex relationships between time and perception or between time and consciousness in general (Heidegger, 1927/2006; Merleau-Ponty, 1964). Here, we focus on just one particular temporal characteristic of perception that is relatively typical of at least first-time aesthetic experiences of each particular object of art: the experience of unexpectedness or the related feelings of surprise on the side of the perceiver. This experience is a direct consequence of the novelty, inventiveness, or ingenuity reflected in many works of art that we emphasized above. Crucially, the experience of unexpectedness entails that perception is a process at least partly related to anticipation and, thus, is a process temporally directed toward the future (*cf*. Van de Cruys and Wagemans, 2011; Friston, 2012; Clark, 2018). Unexpectedness in aesthetic experiences of art is, therefore, a potential avenue toward art appreciation or admiration, where perceivers understand that their surprise is a direct consequence of the creative act from which a work of art originated. In fact, to the degree that the human ability to conceive of the future and to shape it by means of their creativity is itself a function of consciousness (Kotchoubey, 2018; Dresp-Langley, 2022), art appreciation or experiences of beauty prompted by unexpectedness could be a passive but literal form of “creative empathy” with the artist.

## Conclusion

In the present article, we recommend using art aesthetics for a more systematic description of conscious perception. We identified consciousness as a major research frontier in the natural sciences and advocated a view of conscious perception as a process of attributing conscious content to things (or objects) and media. We suggest that this process could exhaustively and successfully be studied by using art perception.

## Data Availability Statement

The original contributions presented in the study are included in the article/supplementary material, further inquiries can be directed to the corresponding author.

## Author Contributions

UA, MP, CQ, MFP, and HL jointly designed the general idea and outline of the manuscript. UA created a first draft. MP, CQ, MFP, and HL revised the manuscript. All authors contributed to the article and approved the submitted version.

## Conflict of Interest

The authors declare that the research was conducted in the absence of any commercial or financial relationships that could be construed as a potential conflict of interest.

## Publisher’s Note

All claims expressed in this article are solely those of the authors and do not necessarily represent those of their affiliated organizations, or those of the publisher, the editors and the reviewers. Any product that may be evaluated in this article, or claim that may be made by its manufacturer, is not guaranteed or endorsed by the publisher.

## Footnotes

^1^In this paper, while we use the term “art,” we do not imply an ontological distinction from other objects or life experiences. People may encounter/react to the environment using a number of general processing sequences that are not confined to any specific stimulus. Indeed, art only has meaning in terms of what it means to an individual perceiver him-or herself. That said, we focus on art because this is (1) a culturally derived class of artifact that often does show rather general consensus regarding what “is” or “is not” art, and (2) because “art encounters,” in the colloquial sense of the term, do routinely evoke, and are often designed to evoke, a complex combination of reactions, which have warranted a special branch of research within psychology. There is also a good deal of evidence to suggest that, when people do think they are engaging with “art,” this evokes specific patterns of expectations/responses in a viewer—based on their personal definition and schema, which also raises key issues for the discussion of consciousness.
